# Admission to the kangaroo mother care ward and maternal postpartum depression in Lusaka, Zambia: a randomised controlled trial study protocol

**DOI:** 10.1136/bmjopen-2026-121368

**Published:** 2026-10-26

**Authors:** Albert Manasyan, Jyoti Lakhwani, Herbert Kapesa, J Anitha Menon, Anjali Sharma, Jake M Pry, Catherine Brown, Taniya Tembo, Waldemar A Carlo

**Affiliations:** 1 Centre for Infectious Disease Research in Zambia, Lusaka, Zambia; 2 University of Alabama at Birmingham, Heersink School of Medicine, Birmingham, Alabama, US; 3 Women and Newborn Hospital, University Teaching Hospitals, Lusaka, Zambia; 4 Curtin University Dubai, Dubai, UAE; 5 Public Health Sciences, University of California Davis Department of Public Health Sciences, Davis, California, US

**Keywords:** Depression, Postpartum, Infant, Developmental neurology & neurodisability, Mortality

## Abstract

**Introduction:**

Postpartum depression (PPD) is a serious public health problem affecting 17% of women globally with higher rates (up to 40%) recorded in southern Africa. Admission to the neonatal intensive care unit (NICU) is a risk factor for PPD. The study aims to determine whether initiation of Kangaroo Mother Care (KMC) at the NICU followed by admission to the KMC Ward for continued support for more than 2 days before discharge home reduces maternal PPD and other maternal and infant adverse outcomes in low-resource settings.

**Methods and analysis:**

This study will enrol mother-infant dyads (n=612), fathers (up to n=612) and a purposive subset of mothers (n=24), fathers (n=24) and family members (n=24) who will complete in-depth interviews (IDIs). Mother-infant dyads will be recruited from the NICU at the Women and Newborn Hospital - University Teaching Hospitals in Lusaka, Zambia. Once deemed stable for admission to the KMC Ward as part of step-down care, mother-infant dyads will be randomised to either the control arm where they will remain at the KMC Ward ≤2 days per standard of care or the intervention arm where they will remain at the KMC Ward for >2 days. The primary outcome is maternal PPD at 6–8 weeks postnatal screened using the Edinburgh Postnatal Depression Scale (EPDS) screening tool. IDIs will explore the feasibility and barriers to practising KMC post-discharge from the perspective of the parents and other family members.

**Ethics and dissemination:**

The study was approved by the University of Zambia Biomedical Research Ethics Committee, the National Health Research Authority and the University of Alabama at Birmingham Institutional Review Board. A Data Safety Monitoring Board (DSMB) has been constituted. Dissemination will be via publications in peer-reviewed journals, conferences, social media and revision of national guidelines.

**Trial registration number:**

NCT06545760.

**STRENGTHS AND LIMITATIONS OF THIS STUDY**

- The study is embedded within routine clinical care, enhancing generalisability to similar settings.
- Longitudinal screening of study participants using validated screening tools allows standardised assessment and long-term impact of the intervention on health outcomes.
- In-depth interviews with parents and family members will provide in-depth understanding of the intervention’s feasibility and challenges after hospital discharge.
- The lack of blinding of study participants and healthcare providers is a limitation that may introduce bias.
- Loss to follow-up after hospital discharge and a high rate of infant mortality may affect the completeness of outcome data.

## Introduction

 Postpartum depression (PPD) is a depressive disorder that commences after childbirth and up to the first year of an infant’s life and is characterised by feelings of worthlessness, fatigue, insomnia, decreased functioning, low mood and suicidal thoughts.^1^ The global prevalence of PPD is 17%, affecting approximately one in six newly delivered mothers.^2 3^ Despite global efforts, the prevalence of PPD increased by 18.4%, reaching as high as 40% in southern Africa.^2 4^

Transition to motherhood is associated with emotional distress, including PPD, affecting about 30% of newly delivered women.^5^ PPD negatively impacts productivity, mood and social interactions with others, including children, and may contribute to economic hardship.^4 6–8^ It can lead to unemployment, contributing to persistent poverty or hindering the person from coming out of poverty due to its impact on mood and mental well-being.^9^ Additionally, admission of a neonate to the neonatal intensive care unit (NICU) is a risk factor for postnatal depression with heightened symptoms in comparison to mothers of well neonates.^10–12^ Children born to mothers with PPD have higher rates of morbidity, malnourishment, lower rates of vaccination uptake and neurodevelopmental delays (NDD).^8–13^

Kangaroo Mother Care (KMC), an intervention used in culturally diverse settings globally, is an effective method to provide care and warmth to the newborn through skin-to-skin care.^14 15^ KMC has also been shown to increase exclusive breastfeeding, neonatal weight gain and mother-infant bonding.^16–18^ Furthermore, it is a proven intervention for the reduction of neonatal mortality among preterm babies.^19 20^ Lastly, in a large randomised controlled trial, early KMC reduced levels of maternal depression through community-initiated KMC practices.^21^ However, early KMC for preterm babies requires resources not available in many hospitals in low-resource settings. Thus, there are limited data on the impact of later KMC starting in the NICU and being followed by care in the KMC Ward on maternal PPD.

We are proposing an individual randomised controlled trial to determine the impact of KMC practised at the NICU, followed by in the KMC Ward, on maternal PPD at the Women and Newborn Hospital - University Teaching Hospitals (WNH-UTH) in Lusaka, Zambia. The study aims to determine whether initiation of KMC at the NICU, followed by admission to the KMC Ward for continued support for more than 2 days before discharge home, reduces maternal PPD and other maternal and infant adverse outcomes in low-resource settings.

## Aim and objectives

### Aim

To determine whether initiation of KMC at the NICU, followed by admission to the KMC Ward for continued support for more than 2 days before discharge home, reduces maternal PPD and other maternal and infant adverse outcomes in low-resource settings. We hypothesise that the practice of NICU-based KMC, followed by admission of the mother-infant (preterm) dyads to the KMC Ward for more than 2 days will reduce the risk of maternal PPD as compared with NICU-based KMC followed by a brief admission of the mother-infant dyad to the KMC Ward for up to 2 days.

### Objectives

Objective 1: To determine the effect of admission of mother-infant dyads to the KMC Ward following NICU for more than 2 days on maternal PPD at 2 weeks, 6–8 weeks, 6 months and 12 months.

Objective 2: To determine the effect of admission of mother-infant dyads to the KMC Ward on infants’ developmental outcomes at 6-, 12- and 18 months of age.

Objective 3: To characterise the feasibility and barriers to uptake of KMC at the hospital and following discharge from the perspective of parents and other family members.

Objective 4: To determine the prevalence of paternal depression.

Objective 5: To estimate the cost and effectiveness of admission of low birth weight infants to the KMC Ward.

## Methods and analysis

### Study design

We are proposing a single-site individual randomised controlled trial with a 1:1 parallel allocation of newly delivered women whose preterm newborns have been admitted to the WNH-UTH NICU with admission to the KMC Ward for up to 2 days (control) or with admission to the KMC Ward for more than 2 days (intervention) before discharge (figure 1). Protocol V.1.2 dated 23 January 2025.

**Figure 1:**
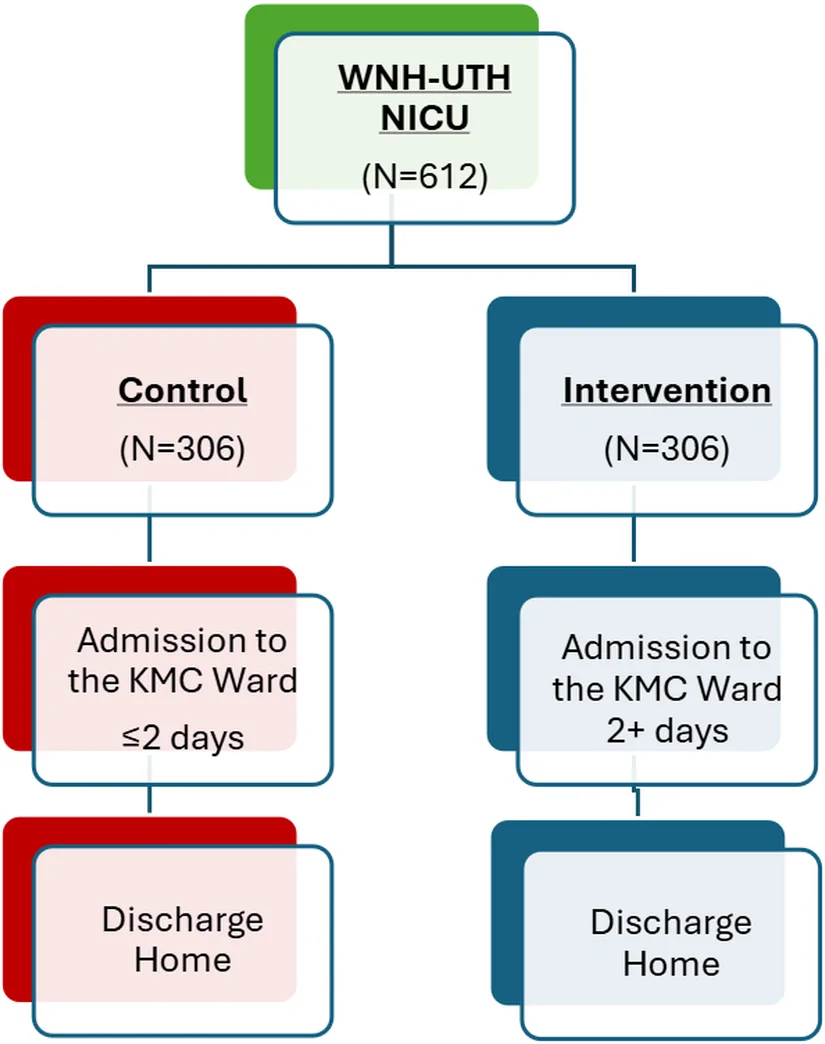
Study arms. KMC, Kangaroo Mother Care; NICU, neonatal intensive care unit; UTH, University Teaching Hospitals; WNH, University Teaching Hospitals.

### Setting

We will implement the study at the WNH-UTH in Lusaka, Zambia. The WNH-UTH records around 10 000 deliveries per year, has a 60-bed space NICU admitting around 4500 newborns per year, a 26-bed KMC Ward, and a Psychiatry Unit. Participants will be enrolled in the NICU and followed up to 18 months postpartum for scheduled screening (figure 2).

**Figure 2:**
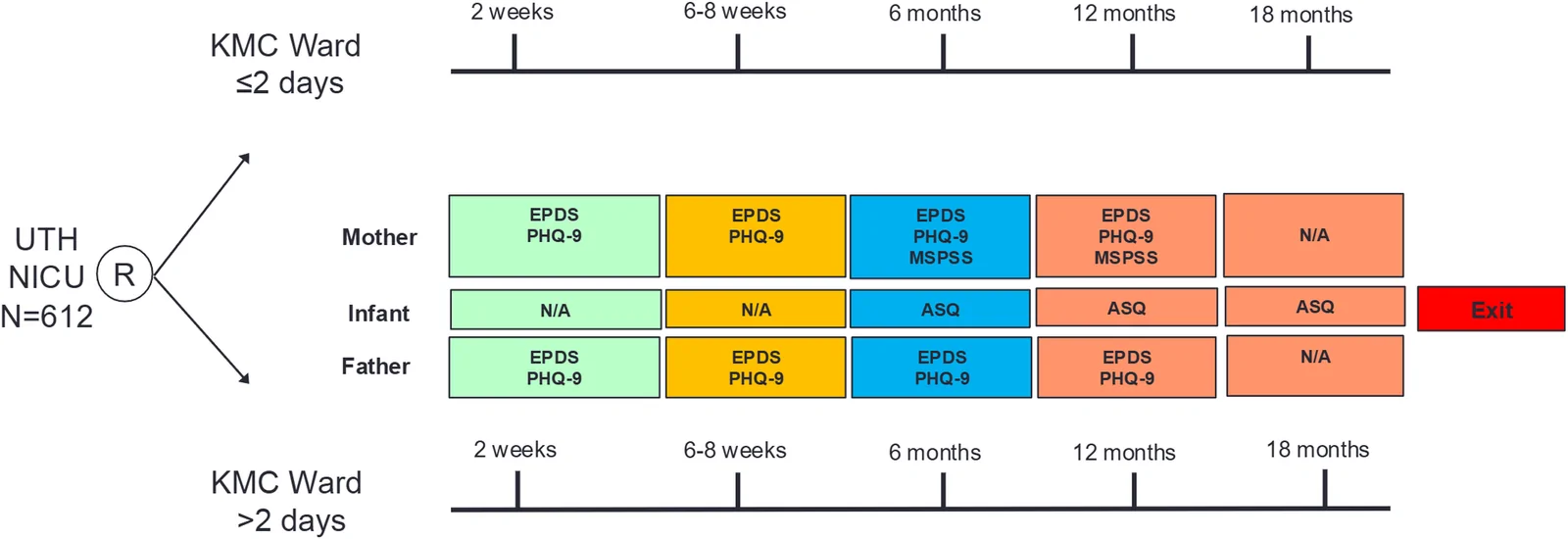
Study assessments and timeline. ASQ, Ages and Stages Questionnaire; EPDS, Edinburgh Postnatal Depression Scale; KMC, Kangaroo Mother Care; MSPSS, Multidimensional Scale of Perceived Social Support; NICU, neonatal intensive care unit; PHQ-9, Patient Health Questionnaire 9; UTH, University Teaching Hospitals.

### Study participants

Newly delivered mothers whose preterm newborns are admitted to the WNH-UTH NICU and those who meet all the eligibility criteria will be approached and consented (written consent) for study participation by a research assistant (RA) (Objectives #1-2 & #5). Similarly, for in-depth interviews (IDIs), parents (mothers and fathers) and trusted family members of the parents enrolled in the study will be approached separately and consented (written consent) for study participation (Objective #3). Lastly, fathers of all newborns enrolled in the study will also be approached and consented (written consent) for study enrolment (Objective #4). All study participants 18+years of age will provide written consent in either English, Nyanja or Bemba. All study participants who are between ages 16–17 will provide written assent after their parents have provided written consent in one of their preferred languages. The consent forms will be made available as online supplemental material. The following inclusion and exclusion criteria will be used for the enrolment of study participants for each study objective (table 1).

**Table 1:** Study participant eligibility criteria

	Eligibility criteria
Objectives #1, #2, #5:	Inclusion criteria: Newborns who are - birth weight between 1,000 g–2,000 g Admitted to the WNH-UTH NICU>48 hours ago Stable preterm eligible for receiving KMC at the KMC Ward and their mothers who are - 16+ years of age Residing within Lusaka Province with no intentions to relocate in the coming 18 months Exclusion criteria: The mother-newborn dyad is excluded if: Mother is on treatment for depression and/or anxiety Mother does not provide informed consent Parent(s) of underage mothers (16–17 years of age) have not provided consent for their participation in the study
Objectives #3:	Inclusion criteria: Parents (mothers and fathers) whose newborn has been enrolled in the study Trusted family member or friend of the mother whose newborn is enrolled in the study 16+ years of age (mothers and fathers) 18+ years of age (family member) Exclusion criteria: Family members or parents who do not consent to study participation
Objectives #4:	Inclusion criteria: Fathers whose newborn has been enrolled in the study 16+years of age Exclusion criteria: Fathers who are on treatment for depression and/or anxiety Fathers who did not provide informed consent Underage fathers (16–17 years of age) whose parent(s) have not provided consent for their participation in the study

KMC, Kangaroo Mother Care; NICU, neonatal intensive care unit; WNH-UTH, Women and Newborn Hospital-University Teaching Hospitals.

### Recruitment

Potential study participants (mother-newborn pairs) will undergo screening, consenting and enrolment at the WNH-UTH NICU. Once deemed clinically stable at the NICU by the treating physician, the newborn will be randomised to either the control or intervention study arms (figure 1) before they are admitted to the KMC Ward as part of routine step-down care. Per standard of care, newborns admitted to the NICU receive intermittent KMC followed by continuous KMC at the KMC Ward where they remain for a minimum of 24 hours before being discharged home. Criteria used to discharge a newborn home from the KMC Ward include the KMC scoring chart, clinical stability of the newborn and the newborn’s weight.

For Aims #1-3, mothers will be informed about the study by study-hired RA. Interested mothers who meet the study eligibility criteria described above will provide written consent. The study RA will also inquire about their willingness to participate in IDIs if selected and to identify a trusted family member or friend to include in IDIs. Randomisation will occur once the newborn is deemed clinically stable at the NICU by the treating physician for admission to the KMC Ward. If, on the planned day of discharge from the NICU or the KMC Ward, the treating physician determines that the newborn requires continued stay based on their clinical condition, the newborn, irrespective of the study arm, will remain at the hospital until the treating physician determines that discharge is appropriate.

For objective #3, study staff trained in qualitative research will approach study participants from study aims #1 and #4 during their 12 month visit or by phone and confirm their interest for study participation under study objective #3. Those interested will provide additional written consent. Additionally, the same staff will approach trusted family members, identified through the mother and father, for participation in the IDIs, who will also be asked to provide written consent. Following enrolment, all study participants will undergo IDIs in a safe and secure location of their choice. The RA will conduct up to 72 IDIs, comprised of 24 purposively selected mothers from Aims #1 (control arm - 12; intervention arm - 12), 24 purposively selected fathers from Aims #4 (control arm – 12; intervention arm - 12) and 24 trusted family members (family member: control arm – 12; intervention arm - 12) per group. Whenever possible, the mother, father and family member will be related to the same infant. IDIs will last approximately 1 hour, and they will be conducted in the preferred language of the consented study participant.

For objective #4, consented mothers will be approached for permission to contact their partners for study enrolment. Those who agree will be asked to provide their partner’s contact information. Reasons for those who decline to provide contact information will be collected. The study RA will contact the partners to inform them about the study and, if interested, they will provide written consent either in person (during their upcoming hospital visit) or over the phone.

For objective #5, we aim to identify major cost centres, quantify all resources and attach a value to the resources required to screen for PPD women whose infants are randomised to be admitted to the KMC Ward. The ingredient-based approach will be applied to estimate the average cost to the health service provider and households for each admitted case. This process will help to determine the unit cost for each cost centre. Costs to be identified include those associated with providing healthcare, costs for patients and their families, to be collected from consenting study participants, productivity costs (opportunity and overhead) and willingness to pay for additional services. We will categorise costs as fixed or variable and direct or indirect costs. Direct costs are the value of all resources consumed in obtaining medical treatment. Direct costs are further subdivided into direct medical and direct non-medical costs. Direct medical costs are the values of resources used for medical treatment such as the costs of drugs, diagnostics, procedures, laboratory tests, supplies, equipment, staff time, allowances and salaries, hospitalisation, outpatient visits and overheads. Direct non-medical costs are the expenses incurred in obtaining medical treatment, such as transportation, food and lodging. Indirect costs are the economic losses borne by the household due to admission to the KMC. They will include 1) the patient and his caretakers’ loss of income/production; substitute labourers’ net loss of income/production; 2) productivity losses due to forgone non-market activities such as school, housework and childcare. Whenever possible, costs borne by households before reaching the hospital for KMC services will be gathered. The cost of one diagnosed case of PPD will be the sum of the costs of all services used for clinical treatment (and diagnosis) plus a fraction of buildings and overhead costs of running the health facility.

For aims #1-2 and #4, there will be a total of up to eight study visits for all study participants and their infants (control and intervention arms) throughout the study. Of these, three will take place while mother-infant dyads are admitted to the hospital and four following their discharge, which are aligned with the postnatal care visits for the infant. The last–eighth–study visit will combine the last clinical screening and study exit interview for all study participants—mother, father, infant (table 2).

**Table 2:** Study visitation schedule

Visit number	Visit name	Study procedures	Clinical screening
0	Eligibility	Pre-screening	N/A
1	Enrollment	Informed consent demographic data	N/A
2	NICU randomisation	Randomisation Data	N/A
3	Hospital discharge (KMC Ward)	Demographic data	Newborn: clinical data
4*	Follow up #1 (2 weeks)	Depression	Mother: EPDS & PHQ-9 Newborn: N/A Father: EPDS & PHQ-9
5	Follow up #2 (6–8 weeks)	Depression	Mother: EPDS & PHQ-9 Newborn: N/A Father: EPDS & PHQ-9
6	Follow up #3 (6 months)	Developmental outcomes and depression	Mother: EPDS, PHQ-9 & MSPSS Newborn: ASQ-3 Father: EPDS & PHQ-9
7	Follow up #4 (12 months)	Developmental outcomes and depression	Mother: EPDS, PHQ-9 & MSPSS Newborn: ASQ-3 Father: EPDS & PHQ-9
8	Follow up #5 (18 months) Study exit	Developmental outcomes	Newborn: ASQ-3

* Some study participants may undergo the fourth visit while still admitted to the hospital (KMC Ward).

ASQ-3, Ages and Stages Questionnaire third Edition; EPDS, Edinburgh Postnatal Depression Scale; KMC, Kangaroo Mother Care; MSPSS, Multidimensional Scale of Perceived Social Support; NICU, neonatal intensive care unit; PHQ-9, Patient Health Questionnaire - 9.

### Retention and follow-up

Strategies to promote participant retention will include obtaining multiple contact details, reminder phone calls prior to follow-up visits and aligning follow-up assessments with routine postnatal care visits where feasible. Efforts will be made to minimise loss to follow-up through flexible scheduling and, where appropriate, telephone-based assessments.

Outcome data will be collected for all participants regardless of adherence to the assigned intervention. Participants who discontinue or deviate from the intervention will be encouraged to complete follow-up assessments, including the primary outcome measure. Reasons for withdrawal or loss to follow-up will be documented.

### Randomization

Participants will be randomised in a 1:1 ratio after the newborn is confirmed to be clinically stable, defined as being off oxygen support and intravenous fluids and feeding well and therefore eligible for admission to the KMC ward. The random allocation sequence will be generated by an independent statistician before trial commencement using block randomisation with variable block sizes and study-specific identification numbers. No stratification will be used. Participant enrolment will be conducted by the RA, who will assign participants to study groups only after eligibility has been confirmed and written informed consent obtained. Allocation concealment will be ensured using sequentially numbered, opaque, sealed envelopes prepared in advance from the randomisation list and opened only after participant enrolment. Study participants will be randomised to one of the following two study arms using block randomisation (figure 1):

*Control Arm:* Study participants randomised to the control arm will be admitted to the KMC Ward for up to 2 days before discharge home by the treating physician using the KMC Ward discharge criteria. During the stay at the KMC Ward, per national guidelines, mothers will be guided on KMC, including breastfeeding, counselled on the importance of continuous KMC and encouraged to continue practising KMC at the KMC Ward and while at home.

*Intervention Arm:* Study participants randomised to the intervention arm will be admitted to the KMC Ward, where they will stay until deemed ready for discharge home, after a minimum of 2 days, by the treating physician using the KMC Ward discharge criteria. on admission to the KMC Ward, mothers will be guided on KMC, including breastfeeding, counselled on the importance of continuous KMC and encouraged to continue practising KMC while at the KMC Ward and at home.

Participants will continue to receive standard postpartum care as per national guidelines. No restrictions will be placed on concomitant care during the trial. Due to the nature of the intervention, participants, care providers and study staff cannot be blinded to group allocation. Outcome assessment will not be blinded. However, the Data Safety Monitoring Board (DSMB) will remain blinded to group allocation during interim reviews where feasible.

### Outcomes

Measurement of study outcomes will be performed at 2 weeks, 6–8 weeks, 6 months and 12 months for mothers and fathers and, for infants, an additional follow-up at 18 months. The study outcomes are detailed below, according to the study objectives (table 3).

**Table 3:** Study outcomes

Study objective	Outcomes
Objective #1	**Primary outcome:** Maternal PPD (6–8 weeks) **Secondary outcome:** Maternal PPD (2 weeks, 6 months and 12 months) Duration of KMC (at the NICU, KMC Ward and at home) Breastfeeding practices, including timing of initiation and duration Barriers to KMC initiation
Objective #2	Neurodevelopmental outcomes at 6, 12 and 18 months of life Weight gain (at NICU and following hospital discharge) Anthropometric measurement All-cause 28-day neonatal mortality Neonatal morbidity Hospital readmission Infant mortality Infant morbidity (sepsis, diarrhoea, respiratory etc.) Vaccination adherence
Objective #3	Barriers and facilitators to practising KMC at home Social-cultural adaptation of KMC
Objective #4	Paternal PPD (2 weeks, 6–8 weeks, 6 months and 12 months) Time spent with newborn
Objective #5	Government cost for admission to the KMC Ward Government cost for readmission to the NICU following randomisation Government costs associated with hospital readmissions within 12 months of life Out-of-pocket family costs associated with hospital readmission, morbidity and mortality

KMC, Kangaroo Mother Care; NICU, neonatal intensive care unit; PPD, postpartum depression.

### Safety

Given the non-invasive and low-risk nature of the intervention, no physical adverse events are anticipated. However, psychological harms, including worsening depressive symptoms or the emergence of suicidal ideation, will be monitored. These will be assessed systematically at each follow-up visit using a validated screening tool described below. Participants identified with elevated scores or any indication of self-harm risk will be referred for further clinical evaluation and appropriate mental health support according to local standard of care. All adverse events will be documented and reported in accordance with study procedures. No additional post-trial care is anticipated beyond standard clinical services. No specific compensation is allocated for participation in the trial. However, participants who experience harm as a result of study participation will receive appropriate medical care in accordance with local institutional policies.

### Assessment

All parents will be screened for PPD (Edinburgh Postnatal Depression Scale (EPDS)), depression (PHQ-9), social support (Multidimensional Scale of Perceived Social Support (MSPSS)) up to 12 months and all infants for NDDs (Ages and Stages Questionnaire third Edition [ASQ-3]) up to 18 months of life, according to the timelines described under figure 2. The study staff will be trained in the use of the screening tools by a master trainer.

Participants who screen positive for PPD or depression using the EPDS or PHQ-9 screening tools, respectively, will be referred to the Psychiatry Department at UTH for further assessment and treatment. Furthermore, infants with delayed milestones will be referred to the Neurology clinic at WNH-UTH. Detailed description of the proposed screening tools is as follows:

### EPDS screening

All mothers and fathers will undergo screening for PPD using the EPDS screening tool postnatally, at four time points: 2 weeks, 6–8 weeks, 6 months and 12 months. This 10-question screening tool is a low-cost and simple screening tool that has been validated for use among pregnant women in LMICs.^22^ It can be used for screening of perinatal depression, beginning from the antenatal period up to 6–8 weeks postnatal. Each question is scored between 0 and 3, the highest cumulative score being 30. Mothers and fathers will be screened for PPD at four time points: 2 weeks, 6–8 weeks, 6 months and 12 months. Mothers who score ≥11 and fathers who score ≥9 will be categorised as having possible PPD and will be linked to care at the WNH-UTH Psychiatry Unit.

### PHQ-9 screening

PHQ-9 is a 9-question patient-administered screening tool for the assessment of depression among mothers and fathers.^23^ The tool has been used in many LMICs, including Zambia.^24^ Mothers and fathers enrolled in the study will be screened for depression using the PHQ-9 screening tool at four time points: 2 weeks, 6–8 weeks, 6 months and 12 months.

### MSPSS

MSPSS is a tool for screening of perceived social support from family, friends and significant other.^25 26^ A brief and easy-to-follow self-report measure, the MPSS is a 12-question tool using a 5-point Likert scale (0=strongly disagree, 5=strongly agree). It uses positive and negative phrasing to limit the response set bias of agreement. Mothers enrolled in the study will be screened using the MSPSS screening tool at 6- and 12-months following enrolment.

### ASQ-3 screening

ASQ is a low-cost, easy to use screening tool that measures the development of young children via screening of 5 domains – communication, problem solving, gross motor, fine motor and personal-social. The screening tool is used between the ages of 1 month and five and a half years and takes around 15 min to complete.^27^ All newborns admitted in this study will be screened for developmental delays using the ASQ tool at 6-, 12- and 18 months of life. The cut-off score for the ASQ-3 tool differs based on each domain and age and are detailed in the screening tool.

### Sample size

A total of 612 study participants will be enrolled in the study for the primary outcomes drawn from a population of mothers whose newborns are admitted to WNH-UTH NICU (Objective #1). Based on our previous study, the control group proportion (P_2_) is assumed to be 0.28. To detect a proportion difference (P_1_ – P_2_) of −0.10 (or P_1_ of 0.18) with 80% power, the number of subjects needed will be 275 in Group 1 (treatment) and 275 in Group 2 (control). Adjusting for a 10% loss to follow-up gives 306 per group, totalling 612 subjects. The sample size was computed using PASS 2023, V.23.0.2.

The sample size for the secondary study outcomes is as follows. For the screening of developmental delays (Objective #2), we will screen infants born to mothers enrolled under Objective #1. Due to infant mortality, we will screen up to n=612 infants for Objective 2. For Objective #3, we will select a purposeful sample of mothers (up to n=24), fathers (up to n=24) and family members (up to n=24) to undergo IDIs. For Objective #4, we will screen fathers of the newborns enrolled in the study. Given that some mothers may not have partners or that some fathers may decline study enrolment, we aim to enrol up to n=612 fathers. Lastly, mothers from the same cohort described above will be approached to collect data for study Objective #5. All together, we will enrol up to 1908 study participants from objectives 1 (n=612), objective 2 (up to n=612), objective 3 (up to n=72) and objective 4 (up to n=612).

### Data collection

Data will be collected directly from patient files and from the study participants by trained study staff and entered into a custom-built study database developed by the Research Electronic Data Capture (REDCap) platform using a tablet. Once entered, data will be reviewed by the data coordinator and/or PI for completeness. While admitted to the hospital, mother-infant dyads will be monitored by the study staff and government staff. Following discharge, study participants, including the fathers, will be requested to return to the study site for follow-up visits as described in figure 2.

Monthly monitoring reports (MMRs) will be generated to monitor study participant screening, enrolment, withdrawals or loss to follow-up, study follow-ups and data queries. MMRs will be generated within 1–2 weeks following the end of the previous month to provide the data entry team ample amount of time for data entry. Once generated, the report will be viewed by the study coordinator and PI. Additionally, monthly data audits will be followed utilising across (data inconsistencies across different data forms) and within (data inconsistencies within the same data forms) algorithms for error resolutions.

Qualitative data will be collected through individual IDIs held in a safe location. Interviews will be conducted in the participant’s preferred language, audio-recorded with consent and transcribed verbatim, with translation into English where necessary. Data collection will continue until thematic saturation is reached. Field notes will be maintained to capture contextual factors and non-verbal cues, enhancing the depth and reliability of the qualitative data.

Individual and facility-level costing data will be collected prospectively alongside the study follow-up visits and entered into the study database. At each scheduled visit, participants will complete a structured, interviewer-administered questionnaire designed to capture direct and indirect costs incurred since the previous visit. To minimise recall bias, cost data will be collected at regular intervals using standardised recall periods aligned with the follow-up schedule. Where feasible, participant-reported expenditures will be cross-checked against available receipts or facility records. All costs will be recorded in local currency and later converted to a common reference year using appropriate inflation adjustments.

### Data management

The Centre for Infectious Disease Research in Zambia (CIDRZ) data management system will be the primary warehouse of study data. Direct access to the database server will be restricted to authorised staff, including individuals who are responsible for monitoring and backing up the system. All other access to the database server will be controlled by logical security and occur across a secure network protected by password access and system controls.

Study data will be entered and stored in REDCap, a highly intelligent data management capturing system that ensures accurate and timely data entry and management. Case reporting forms have been designed specifically for this study. The data system will use ‘form look-alike’ screens that include automated range, consistency, verification and ID checks where possible. Fortnightly data reviews will be conducted by site staff for data completeness and accuracy (edit reports) followed by error resolutions. MMRs will be created to monitor patient enrolment for each cohort throughout the study enrolment period. The principal investigator will be responsible for assuring that the data entered into the database is complete, accurate, consistent with the source documents and that entry and updates are performed in a timely manner. Corrections and alterations of data on the database or source documents must be made by the investigator or by the designated person from his/her team and must be dated and signed. The identity of participants will be protected by the removal of any identifiable data before it is transferred to the statistician for analysis. Additionally, no identifiable data will be shared when disseminating study findings.

### Data sharing

After completion of the study and publication of the primary results, de-identified individual participant-level data will be available from the corresponding author on reasonable request. The study protocol will be made available as online supplemental material or through an appropriate repository. Requests for access to participant-level data will be reviewed by the study investigators and will be subject to applicable ethical, regulatory and institutional requirements.

### Statistical analysis

**Objective #1:** The primary analysis will compare the proportion of maternal PPD at 6–8 weeks postnatal between the two arms using a two-sided, two-sample Z-test with unpooled variance. We will estimate risk ratio (crude and adjusted) using a log-binomial regression model. A likelihood ratio p-value less than 0.05 will be considered statistically significant. We will conduct a multiple regression analysis to predict depression during the postpartum period using EPDS screening results collected prior to randomisation and during follow up visits. Data will be dichotomised into those with depressive symptoms and those without and further grouped into those with anxiety and feelings of self-harm. Efforts will be made to minimise missing data through active follow-up. The primary analysis will be conducted using available data under the intention-to-treat principle. Sensitivity analyses will be performed to assess the impact of missing data, including complete-case analysis and, where appropriate, multiple imputation under the assumption that data are missing at random. All randomised participants will be included in the primary analysis according to the group to which they were assigned, regardless of adherence to the intervention or protocol deviations (intention-to-treat principle). A per-protocol analysis may also be conducted including only participants who adhered to the assigned intervention, as a sensitivity analysis.

**Objective #2:** Using a log-binomial regression model, we will estimate the effect of the intervention on the risk of NDDs, adjusting for potential confounders. Children at risk of NDD will be classified using scores obtained from the five ASQ-3 domains compared to the established cut-off. Each of the five domains (communication, problem-solving, gross motor, fine motor and personal and social domains) are scored between 0 and 60 with higher scores being indicative that the child’s development is on schedule. Binary secondary outcomes will be analysed in an analogous way. However, continuous secondary outcomes will be analysed using linear regression model, adjusted for important confounders.

**Objective #3:** Qualitative data will be analysed using framework analysis to capture the perspectives of mothers, fathers and trusted person. Deductive coding will be applied using a priori codes derived from literature. Analysis will focus on identifying the feasibility and barriers of practising KMC in the hospital and at the household following hospital discharge from the perspective of parents and family members with comparisons conducted by study arm. IDIs will be audio-recorded and transcribed directly into English and uploaded to NVivo 9.0 (QSR International, Australia) for data management and analysis. Analysis will begin with the familiarisation process that will involve reading the transcripts, identifying key meanings related to the facilitators and barriers and coding them under the appropriate pre-defined codes to identify themes. Themes will be indexed and charted to summarise insights by participant type, allowing for comparisons across and within study arms to identify and interpret variation and connections between the household/community barriers/facilitators of practising KMC at home.

**Objective #4:** A similar analysis approach described above under Objective #1 will be used to determine the overall prevalence of paternal depression using EPDS and PHQ-9 at 6–8 weeks and 12 months postpartum and prevalence within each arm. Furthermore, we will look at the correlation between maternal and paternal PPD overall at 12 months postpartum.

**Objective #5:** An economic evaluation will be conducted from a societal perspective to determine the cost-effectiveness of the extended KMC ward strategy compared with standard care. The analysis will compare the relative cost and health outcomes of the two strategies by randomised stratification—the intervention (one that proposes to screen for PPD among women whose infants are admitted to the KMC ward for >2 days) and the control (women whose infants are admitted to the KMC ward for ≤2 days). Costs will be captured prospectively using an ingredient-based costing approach and categorised into: 1) health system costs (direct medical: eg, hospital stay, staff time, supplies; direct non-medical: overheads; 2) household direct and indirect costs: family transportation, food, productivity losses for mothers, fathers and caregivers and 3) research/programme costs. Data will be sourced from hospital finance records and structured questionnaires at discharge and follow-up visits. Clinical effectiveness will be measured using the trial’s primary outcome (the difference in PPD incidence at 6–8 weeks postpartum), with effect sizes estimated as risk differences and ratios, adjusted for confounders. The primary economic outcome will be the incremental cost-effectiveness ratio (ICER), calculated as the difference in total costs divided by the difference in health effects between the two arms. Effects will be expressed as cost per PPD case averted, cost per infant death averted and cost per disability life year (DALY) averted. DALYs will incorporate years of life lost and years with disability, using global burden of disease weights and the country-specific life expectancy tables from the WHO, with future costs and effects discounted at 3%. Deterministic and probabilistic sensitivity analyses will be performed to assess the robustness of the ICER by varying key parameters (eg, hospital stay per day, PPD prevalence etc.). This approach will quantify the incremental resources needed to achieve each additional health gain, providing evidence critical for guiding resource allocation, additional investment required to achieve one additional unit of health benefit and maternal-child health policy in low- and middle-income countries.

Prespecified additional analyses will include sensitivity analyses to assess the robustness of the primary findings, including analyses restricted to participants with complete outcome data and, where appropriate, alternative approaches to handling missing data. No formal subgroup analyses are planned. Any additional analyses not specified a priori will be considered exploratory.

### Data safety and monitoring board

The trial is coordinated by the CIDRZ, which serves as the coordinating site and is responsible for overall study management, data collection and site supervision. A dedicated data management team oversees data quality, storage and security. The study investigators are responsible for trial conduct and oversight.

A DSMB will be assigned to the study and is tasked to safeguard the interests of the study participants and to assist and advise the PI to protect the validity and credibility of the study. We recommend that the DSMB meet formally to review interim safety, efficacy and futility, as well as other aspects of accruing data (such as enrolment, data completeness and protocol compliance), three times during the study, after roughly 25%, 50% and 75% of subjects complete the 6–8 week follow-up. DSMB reports will be blinded, with study arms labelled as group A and group B. However, the DSMB may request to be unblinded to perform their duties. The DSMB will have full authority to suspend enrolment or stop the study altogether due to patient safety. The DSMB will debrief with the study investigators regarding their findings. A report will be written following this feedback, and the research team will respond and react to recommendations made by the DSMB.

### Patient and public involvement

People living with PPD were not involved in the development of the study protocol. However, we plan to involve mothers and fathers with and without PPD in some aspects of the qualitative data analysis. Lastly, we will involve mothers and fathers living with PPD in the study dissemination plans.

### Ethics and dissemination

The study was approved by the University of Zambia Biomedical Research Ethics Committee (#5089-2024), the National Health Research Authority (#NHREB/NHRA-1177/13/05/2024) and the University of Alabama at Birmingham Institutional Review Board (IRB-300012872).

The findings of this trial will be disseminated through peer-reviewed publications and presentations at scientific conferences. Results will also be shared with participating healthcare facilities and relevant stakeholders in Zambia. A summary of findings will be made available to study participants on request and through community engagement platforms. Trial results will be reported in the clinical trial registry in accordance with applicable guidelines. Where appropriate, plain language summaries will be developed to facilitate dissemination to non-specialist audiences. Patients and the public were not involved in the design, conduct, reporting or dissemination plans of this study.

## Supplementary material

10.1136/bmjopen-2026-121368 online supplemental file 1

## Data Availability

Data are available upon reasonable request. No data are available.
