# Supplementary material for: Admission to the kangaroo mother care ward and maternal postpartum depression in Lusaka, Zambia: a randomised controlled trial study protocol
Source: BMJ Open. 2026 Oct 26;16(10):e121368. doi: 10.1136/bmjopen-2026-121368 (PMC13711544; doi:10.1136/bmjopen-2026-121368)
Supplement: Supplementary file 1 [file bmjopen-2026-121368.reviewer_comments.pdf]

PEER REVIEW HISTORY

BMJ Open publishes all reviews undertaken for accepted manuscripts. Reviewers are asked to complete a checklist review form and are provided with free text boxes to elaborate on their assessment. These free text comments are reproduced below.

ARTICLE DETAILS

Title (Provisional)

Admission to the Kangaroo Mother Care Ward and Maternal Postpartum Depression in Lusaka, Zambia: A Randomized Controlled Trial Study Protocol

Authors

Manasyan, Albert; Lakhwani, Jyoti; Kapesa, Herbert; Menon, J. Anitha; Sharma, Anjali; Pry, Jake M; Brown, Catherine; Tembo, Taniya; Carlo, Waldemar A

VERSION 1 - REVIEW

**Reviewer**	**1**
**Name**	**Hashimoto, Kenji**
**Affiliation**	**Chiba University Center for Forensic Mental Health**
**Date**	**21-May-2026**
**COI**	**None**

In the study, the authors aim to determine whether initiation of Kangaroo Mother Care (KMC) at the NICU, followed by admission to the KMC Ward for continued support for more than 2 days before discharge home, reduces maternal PPD and other maternal and infant adverse outcomes in low-resource settings. This study will enroll mother-infant dyads (n=612), fathers (up to n=612), and a purposive subset of mothers (n=24), fathers (n=24), and family members (n=24) who will complete in-depth interviews (IDIs). Mother-infant dyads will be recruited from the NICU at the Women and Newborn Hospital - University Teaching Hospitals in Lusaka, Zambia. Once deemed stable for admission to the KMC Ward as part of step-down care, mother-infant dyads will be randomized to either the control arm, where they will remain at the KMC Ward ≤ 2-day stay per standard of care, or the intervention arm, where they will remain at the KMC Ward for >2 days. The primary outcome is maternal PPD at 6-8 weeks postnatal, screened using the Edinburgh Postnatal Depression Scale (EPDS) screening tool. IDIs will explore the feasibility and barriers to practicing KMC post-discharge from the perspective of the parents and other family members. Additionally, the study will determine the cost implications of the proposed intervention, the barriers and feasibility for practicing KMC from the perspective of mothers, fathers, and family members following hospital discharge, and the prevalence of paternal

postpartum depression. This protocol is well conducted, and this article is well written. I have no concerns about the article.

**Reviewer**	**2**
**Name**	**Ahmed, Sheikh**
**Affiliation**	**The Aga Khan University, Obstetrics and Gynecology**
**Date**	**11-Aug-2026**
**COI**	**None**

Thank you for the opportunity to review this well-written protocol. The study is relevant and clearly presented. I have a few minor comments for the authors' consideration:

1. Study Participants/Ethical Considerations: Please clarify the consent process for underage participants, including parental/guardian consent and participant assent, where applicable.

2. Eligibility Criteria: Please explicitly define the eligibility criteria for the control group, particularly regarding infants remaining in the KMC ward for ≤2 days, to ensure consistency with the intervention criteria (>48 hours).

3. Lines 419–422: The description of the control group appears unclear. As the control group comprises women whose infants are admitted to the KMC ward for ≤2 days, this should be stated explicitly.

4. Data Management: Please clarify whether personal or identifiable information will be collected and describe the procedures for its collection, storage, access, sharing, and retention to ensure confidentiality throughout and after the trial.

VERSION 1 - AUTHOR RESPONSE

Reviewer: 1
Dr. Kenji Hashimoto, Chiba University Center for Forensic Mental Health
Comments to the Author:

In the study, the authors aim to determine whether initiation of Kangaroo Mother Care (KMC) at the NICU, followed by admission to the KMC Ward for continued support for more than 2 days before discharge home, reduces maternal PPD and other maternal and infant adverse outcomes in low-resource settings. This study will enroll mother-infant dyads (n=612), fathers (up to n=612), and a purposive subset of mothers (n=24), fathers (n=24), and family members (n=24) who will complete in-depth interviews (IDIs). Mother-infant dyads will be recruited from the NICU at the Women and Newborn Hospital - University Teaching Hospitals in Lusaka, Zambia. Once deemed stable for admission to the KMC Ward as part of step-down care, mother-infant dyads will be randomized to either the control arm, where they will remain at the KMC Ward ≤ 2-day stay per standard of care, or the intervention arm, where they will remain at the KMC Ward for >2 days. The primary outcome is maternal PPD

at 6-8 weeks postnatal, screened using the Edinburgh Postnatal Depression Scale (EPDS) screening tool. IDIs will explore the feasibility and barriers to practicing KMC post-discharge from the perspective of the parents and other family members. Additionally, the study will determine the cost implications of the proposed intervention, the barriers and feasibility for practicing KMC from the perspective of mothers, fathers, and family members following hospital discharge, and the prevalence of paternal postpartum depression. This protocol is well conducted, and this article is well written. I have no concerns about the article.

> **Response:** Thank you for the encouraging feedback.

Reviewer: 2
Dr. Sheikh Ahmed, The Aga Khan University
Comments to the Author:

Thank you for the opportunity to review this well-written protocol. The study is relevant and clearly presented. I have a few minor comments for the authors' consideration:

Comment #13: Study Participants/Ethical Considerations: Please clarify the consent process for underage participants, including parental/guardian consent and participant assent, where applicable.

> **Response:** Thank you for your comment. We have revised the paragraph (lines 164 - 167) to indicate the consent/assent process for the study participants based on their age:
>
> All study participants 18+ years of age will provide written consent in either English, Nyanja, or Bemba. All study participants who are between ages 16-17 will provide written assent after their parents have provided written consent in one of their preferred languages.

Comment #14: Eligibility Criteria: Please explicitly define the eligibility criteria for the control group, particularly regarding infants remaining in the KMC ward for ≤2 days, to ensure consistency with the intervention criteria (>48 hours).

> **Response:** Thank you for your comment. We have revised this section to clarify that the statement regarding ">48 hours" pertains to patients being admitted to the NICU more than 48 hours ago, which is different from the criteria in the randomization plan. This has been changed in the trial registry, too. The criteria now read (Table 1):
>
> 2. Admitted to the WNH-UTH NICU **>48 hours ago**

Comment #15: Lines 419–422: The description of the control group appears unclear. As the control group comprises women whose infants are admitted to the KMC ward for ≤2 days, this should be stated explicitly.

> **Response:** Thank you for noting this. We have revised this section to reflect the following (lines 495- 496):
>
> The analysis will compare the relative cost and health outcomes of the two strategies

by randomized stratification– the intervention (one that proposes to screen for PPD among women whose infants are admitted to the KMC ward for >2 days) and the control (**women whose infants are admitted to the KMC ward for ≤2 days).**

Comment #16: Please clarify whether personal or identifiable information will be collected and describe the procedures for its collection, storage, access, sharing, and retention to ensure confidentiality throughout and after the trial.

Response: Thank you for the recommendation. The Data Management paragraph has been revised, and an additional paragraph titled "Data Sharing" has been added (Lines 423 – 448):

Data Management (Lines 423 – 442):
The Centre for Infectious Disease Research in Zambia (CIDRZ) data management system will be the primary warehouse of study data. Direct access to the database server will be **restricted to authorised staff,** including individuals who are responsible for monitoring and backing up the system. All other access to the database server will be controlled by logical security and occur across a secure network protected by password access and system controls.
Study data will be **entered and stored in the Research Electronic Data Capture (REDCap) platform,** a highly intelligent data management capturing system that ensures accurate and timely data entry and management. **Case reporting forms have been designed specifically for this study.** The data system will utilize "form look-alike" screens that include automated range, consistency, verification, and ID checks where possible. Fortnightly data reviews will be conducted by site staff for data completeness and accuracy (edit reports) followed by error resolutions. Monthly monitoring reports will be created to monitor patient enrolment for each cohort throughout the study enrolment period. The principal investigator will be responsible for assuring that the data entered into the database is complete, accurate, consistent with the source documents and that entry and updates are performed in a timely manner. Corrections and alterations of data on the database or source documents must be made by the investigator or by the designated person from his/her team and must be dated and signed. **The identity of participants will be protected by the removal of any identifiable data before it is transferred to the statistician for analysis. Additionally, no identifiable data will be shared when disseminating study findings.**

Data Sharing (Lines 443 – 448):
After completion of the study and publication of the primary results, de-identified individual participant-level data will be available from the corresponding author upon reasonable request. The study protocol will be made available as supplementary material or through an appropriate repository. Requests for access to participant-level data will be reviewed by the study investigators and will be subject to applicable ethical, regulatory, and institutional requirements.
