# Supplementary material for: Admission to the kangaroo mother care ward and maternal postpartum depression in Lusaka, Zambia: a randomised controlled trial study protocol
Source: BMJ Open. 2026 Oct 26;16(10):e121368. doi: 10.1136/bmjopen-2026-121368 (PMC13711544; doi:10.1136/bmjopen-2026-121368)
Supplement: Supplementary file 2 [file bmjopen-16-10-s001.docx]

**Admission to the Kangaroo Mother Care Ward and Maternal Postpartum Depression:**

**a Randomized Controlled Trial**

**(Short title: KMC PPD Study)**

**Protocol version 1.2**

**23^rd^ January 2025**

1. Dr Albert Manasyan
2. Prof. Anitha Menon
3. Dr. Jyoti Lakhwani

**Study Sponsor:**

Grand Challenges Canada

Table of Contents

[Investigator Roster	3](#_Toc171940042)

[Abbreviations	4](#_Toc171940043)

[1.0	Introduction	5](#_Toc171940044)

[2.0	Statement of the Problem	6](#_Toc171940045)

[3.0	Rationale/Justification	6](#_Toc171940046)

[4.0	Literature Review	7](#_Toc171940047)

[5.0	Research Question	8](#_Toc171940048)

[6.0	Specific Objectives	8](#_Toc171940049)

[7.0	Methodology	9](#_Toc171940050)

[Study Design	9](#_Toc171940051)

[Study Site	9](#_Toc171940052)

[Study Population	9](#_Toc171940053)

[Sample Size	10](#_Toc171940054)

[Study Procedures	11](#_Toc171940055)

[Randomization	13](#_Toc171940056)

[Description of Clinical Screening	14](#_Toc171940057)

[Data Collection	16](#_Toc171940058)

[Data Analysis	18](#_Toc171940059)

[Dissemination Plan	19](#_Toc171940060)

[8.0	Ethical Considerations	19](#_Toc171940061)

[Potential Risks	20](#_Toc171940062)

[Potential Benefits	20](#_Toc171940063)

[Data Safety and Monitoring Board (DSMB)	20](#_Toc171940064)

[9.0	Timeline	20](#_Toc171940065)

[10.0	Budget	21](#_Toc171940066)

[11.0	Appendix	21](#_Toc171940067)

[References	22](#_Toc171940068)

# Investigator Roster

*Albert Manasyan, MD, MPH*

Principal Investigator

Head of RMNCH Department

Centre for Infectious Disease Research in Zambia

Associate Professor

University of Alabama at Birmingham

*J. Anitha Menon, MA, PhD*

Co-Investigator

Professor of Health Psychology

University of Zambia

*Waldemar A. Carlo, MD*

Co-Investigator

Professor of Pediatrics

University of Alabama at Birmingham

*Jyoti Lakhwani, MD, MPH, MHLM*

Co-Investigator

Neonatology Fellow

Neonatal Intensive Care Unit, University Teaching Hospitals

*Amelia Schuyler, MD*

Co-Investigator

Neonatology Fellow

University of Alabama at Birmingham

*Catherine Brown, MD*

Co-Investigator

Child Neurology Fellow

Cincinnati Children’s Hospital Medical Centre

# Abbreviations

ASQ-3	Ages and Stages Questionnaire 3^rd^ Edition

CIDRZ	Centre for Infectious Disease Research in Zambia

DSMB	Data Safety and Monitoring Board

EPDS	Edinburgh Postnatal Depression Scale

HIV	Human Immunodeficiency Virus

IDI	In-Depth Interview

IRB	International Review Board

KMC	Kangaroo Mother Care

PAS	Pediatric Academy of Societies

PHQ-9	Patient Health Questionnaire - 9

PPD	Postpartum Depression

LMIC	Low- and Middle-Income Country

MoH	Ministry of Health

MMR	Monthly Monitoring Report

NHRA	National Health Research Authority

NICU	Neonatal Intensive Care Unit

NDD	Neurodevelopmental Delays

UNZA BREC	University of Zambia Biomedical Review Ethics Committee

UAB	University of Alabama at Birmingham

UTH	University Teaching Hospitals

WHO	World Health Organization

WNH	Women and Newborn Hospital

# 1.0 Introduction

With less than a decade to achieve Sustainable Development Goal 3.4 of promoting mental health, improving the identification of women with postpartum depression (PPD), reducing the burden, and ensuring their linkage to care is even more crucial than ever.[1] Global prevalence of PPD is 17%, amounting to an estimated 24 million newly delivered mothers worldwide.[2, 3] Despite global efforts, the prevalence of PPD increased by 18.4% with Africa having the highest burden (40-50%).[4, 5] PPD is a depressive disorder that commences after childbirth and up to the first year of an infant’s life, and is characterized by feelings of worthlessness, fatigue, insomnia, decreased functioning, low mood, and even suicidal thoughts.[6] Transition to motherhood is associated with emotional distress, including PPD, affecting about 30% of newly delivered women.[7] A recent review of the magnitude of maternal postpartum depression in Zambia found that 64% of newly delivered mothers have PPD.[8] However, despite this high prevalence, vast majority of mental health related studies in Zambia are linked exclusively to people living with HIV.[9-11]

Postpartum depression has a negative impact on productivity, mood, and social interactions with others, including the children, and economic hardship.[12-15] It can lead to unemployment contributing to poverty or not allowing the person to move out of poverty due to its impact on one's mood and mental wellbeing.[16] Furthermore, admission of a neonate to the NICU is a risk factor for postnatal depression with heightened symptoms in comparison to mothers of well neonates.[17-19] Children born to mothers with PPD have higher rates of morbidity, malnourishment, lower rates of vaccination uptake, and neurodevelopmental delays (NDD).[15, 20]

Kangaroo Mother Care [KMC], an acceptable intervention in low-resource and culturally diverse settings, is an effective method to provide care and warmth to the newborn through skin-to-skin care.[21, 22] KMC has also been shown to contribute to increased exclusive breastfeeding, neonatal weight gain, and mother-infant bonding.[23] Furthermore, it is a proven intervention for the reduction of neonatal mortality among preterm babies.[24] Lastly, KMC is associated with reduced levels of maternal depression through community-initiated KMC practices.[25] However, the impact of KMC on maternal PPD among women whose newborns have been admitted to the NICU and get KMC as part of the dropdown care has not been studied.

Increased physical interaction between the mother and her newborn has been shown to have a protective effect against developing PPD.[26] Furthermore, improvement in maternal PPD rates lead to an improved feeling of self-worth, which in turn will contribute to mother-infant bonding ensuring higher quality of care of the newborn. We are proposing an individual randomized controlled trial to determine the impact of KMC practiced at the NICU followed by in the KMC ward on maternal PPD at the Women and Newborn Hospital - University Teaching Hospitals (WNH-UTH) in Lusaka, Zambia. Our overarching objective is to determine whether initiation of KMC at the NICU followed by admission to the KMC ward for continued support reduces maternal PPD and other maternal and infant adverse outcomes in low-resource settings.

# 2.0 Statement of the Problem

Perinatal depression is a serious public health problem among women, occurring from the first trimester (peripartum depression) up to the first year after delivery (postpartum depression).[6] Maternal PPD not only results in increased maternal morbidity, decreased quality of life, and suicidal thoughts but can also negatively impact the newborn’s health and development leading to growth impairment, non-exclusive breastfeeding, poor adherence to immunization, and hospitalization.[13-15] Furthermore, despite the staggering rates of maternal PPD in LMIC settings, children born to women with PPD are at higher risk of adverse health outcomes including growth and development but lack services and access to evidence-based care for mental wellness.[27] Factors such as mode of delivery and physical separation of the newborn from the mother for a neonatal intensive care unit (NICU) admission where the newborn's overall health and survival are at risk have been found to be risk factors for PPD.[17-19, 26] However, while KMC has been shown to reduce maternal PPD in community settings, its impact on mothers whose preterm newborns have been admitted to the NICU has not been studied to the best of our knowledge.[25]

# 3.0 Rationale/Justification

Postpartum depression has been associated with poor delivery outcomes, breastfeeding practices, malnutrition, etc.[13-15] Unlike in many high-income countries, the identification and treatment of patients with perinatal depression is not prioritized in many low- and middle-income countries.[28] Furthermore, while services exist for the management of maternal PPD in LMICs, the lack of implementation and routine screening throughout all levels of government health facilities leads to both delayed identification and identification of women with already severe PPD.[29] Studies conducted in Zambia have found that up to 64% of newly delivered mothers have PPD.[8] However, despite these rates, PPD screening is not done routinely and majority of mental health related studies in Zambia are linked exclusively to the uptake and adherence to ART services.[9-11] Our intervention builds on a culturally acceptable intervention – Kangaroo Mother Care – that has been embedded in many low-resource settings, which has the potential to reduce maternal PPD, thus reducing the rates and consequences of undiagnosed and untreated maternal PPD due to lack of screening and delayed identification.

Through a secondary analysis of an existing study (UNZA BREC Ref. No. 2512-2021; manuscript under development) that assessed postnatal growth restrictions, we found that 23.0% of mothers discharged from the KMC ward screened positive for PPD. As a follow up step, we determined the prevalence of PPD among newly delivered mothers, grouping the findings based on the required postnatal care of their newborn – admission to the postnatal ward, KMC ward, or NICU (UNZA BREC Ref. No. 3814-2023; manuscript under development). While 20.5% of all mothers had PPD, mothers whose newborns were admitted to the KMC ward after NICU had a significantly lower rate of PPD (21.3%) compared to those directly discharged home directly from the NICU (27.8%) (*p=0.01*) (RR 0.59, 95% CI 0.39-0.88). These study findings have led us to hypothesize that mothers whose newborns were admitted to the NICU may require further support prior to discharge home where they can bond with their newborn in a safe and conducive environment prior to discharge home – at the KMC ward – leading us to propose to determine the effect of admission to the KMC ward through a randomized controlled trial.

# 4.0 Literature Review

Globally, the prevalence of PPD has increased by 18.4% amounting to an estimated 40 million newly delivered mothers diagnosed with PPD worldwide.[2, 30] Africa bears the highest burden - 40-50%.[4, 5] While differences in cultural practices may play a role in the existing inequality high- and low- and middle-income countries, existing institutional biases (e.g., religion, caste) and cultural beliefs (e.g., stigma, seeking support from traditional healers) against women increase the burden of illness in this target population.[5, 31] Cultural beliefs influence health-seeking behaviors, which negatively influence access to appropriate mental health services in LMIC settings.[32] Furthermore, studies have shown a strong association between depression and poverty due to reduced income and recurring healthcare costs for the mother and the newborn.[16, 33] Studies have shown that reduction in maternal PPD can lead to improvement in the following categories:

1. **Personal/newborn** – improvements in the stress levels, mood, and outlook of the mother, which in turn has shown to have a positive impact on the well-being of the newborn, including breastfeeding practices, malnutrition, immunizations, and morbidities such as diarrhea.[13-15]
2. **Community/family** – improved relationships with family members including the partner/husband and the children, including their cognitive development.[15, 20]
3. **Economic** – higher rate of employment by the mother thus reducing the risk of food insecurity at the household, ultimately reducing the health disparities and economic inequity. [12, 34]
4. **Healthcare costs** – reduced short- and long-term (lifetime) costs on both the mother and the newborn borne both by the family and the government due to improved health outcomes of the mother-infant dyad.[35, 36]

The impact of KMC on maternal PPD and infant outcomes has been studied but in community settings.[24, 25] A recent randomized controlled trial done in India showed that community initiated KMC resulted in a 25% reduction in the relative risk of moderate to severe depression measured at 4 weeks postpartum using the Patient Health Questionnaire-9 (PHQ-9) screening tool.[25] However, this study was done among stable newborns weighing 1500 – 2500 grams and may not be applicable to infants <1500 grams.[25] Other quasi-experimental studies conducted in Canada and Iran have shown the positive effect of practicing skin-to-skin on maternal mental health.[37, 38] We found only one observational study conducted in Brazil evaluating the impact of KMC on maternal mental health practiced at the KMC unit.[39] However, KMC was initiated only following NICU discharge and not while commenced while at the NICU as per new World Health Organization (WHO) recommendations. While these studies show the acceptability and effectiveness of KMC in different settings, our literature review found no randomized controlled trials evaluating the effectiveness of admission to the KMC ward following NICU discharge on maternal PPD. Additionally, studies done in high-income countries are not always relevant to low-resource settings due to sociocultural differences and socioeconomic factors confounding mental health issues.

# 5.0 Research Question

Our research questions are:

1. Does admission of mother-infant dyads to the KMC ward following NICU discharge for more than 2 days reduce maternal PPD?
2. Does KMC improve infant neurodevelopmental outcomes at 18 months of age?
3. What are the barriers for mothers to practicing KMC following hospital discharge?
4. What is the prevalence of paternal PPD?
5. Is it cost-effective to admit preterm mother-infant dyads to the KMC ward following NICU discharge?

# 6.0 Specific Objectives

Our overarching **objective** is to determine whether initiation of KMC at the NICU followed by admission to the KMC ward for continued support for more than 2 days before discharge home reduces maternal PPD and other maternal and infant adverse outcomes in low-resource settings. Our **central hypothesis** is that NICU KMC plus admission of mother-infant (preterm) dyads to the KMC ward following NICU discharge for more than 2 days will reduce the risk of maternal PPD as compared to NICU KMC followed by admission of the mother-infant dyad to the KMC ward for up to 2 days.

Our study aims are:

**Aim 1:** To determine the effect of admission of mother-infant dyads to the KMC ward following NICU for more than 2 days on maternal PPD at 2-weeks, 6-8 weeks, 6 months, and 12 months.

**Aim 2:** To determine the effect of admission of mother-infant dyads to the KMC ward on infant’s developmental outcomes at 6-,12-, and 18 months of age.

**Aim 3:** To characterize the feasibility and barriers to uptake of KMC at the hospital and following discharge.

**Aim 4:** To determine the prevalence of paternal depression.

**Aim 5:** To estimate the cost and effectiveness of admission of low birthweight infants to the KMC ward:

a. Estimate costs from a health systems perspective and explore cost drivers of KMC and NICU care for low birthweight newborns
b. Assess out-of-pocket expenditure incurred by households due to admission of low birthweight infants to the KMC and NICU
c. Estimate the incremental cost-effectiveness of screening for postpartum depression among women with low birthweight infants admitted to the KMC compared to the status quo

# 7.0 Methodology

## Study Design

We are proposing an individual randomized controlled trial with a 1:1 parallel allocation of newly delivered women whose preterm newborns have been admitted to the WNH-UTH NICU with admission to the KMC ward for up to 2 days (control) or with admission to the KMC ward for more than 2 days (intervention) prior to discharge home. The study will enroll a total of 1908 study participants from both study arms pulled from 612 mothers (aim #1), 612 infants (aim #2), 72 participants for in-depth interviews (aim #3), and 612 fathers (aim #4).

## Study Site

We are proposing to implement the study at the WNH-UTH in Lusaka, Zambia. The WNH-UTH records around 15,000 deliveries per year, has a 60-bed space NICU admitting around 4500 newborns per year, 26-bed KMC ward admitting around 1200 preterm babies per year, and a Psychiatry Unit (Clinic 6). Participants will be enrolled in the NICU and followed up to 18 months postpartum for scheduled screening (Figure 2).

## Study Population

Newly delivered mothers whose preterm newborns are admitted to the WNH-UTH NICU and those who meet all the inclusion and exclusion criteria will be approached and consented for study participation (Aims #1-2 & #5). For in-depth interviews (IDIs), parents (mothers and fathers) and trusted family members of the parents will be approached and consented for study participation (Aim #3). Lastly, fathers of all newborns enrolled into the study will also be approached for study enrolment (Aim #4). In 2022, WNH-UTH admitted 4,768 NICU and 1,376 KMC admissions. Our previous trial had a consent rate of 90%. Therefore, we are confident that we will enroll our target sample size within 12 months.

**Inclusion and Exclusion Criteria**

The following inclusion and exclusion criteria will be used for enrolment of study participants for each study aim.

Aims #1-2 and #5:

Inclusion criteria: Mothers to newborns who are -

1) Birthweight between 1,000 – 2,000
2) Admitted to the WNH-UTH NICU (>48 hours)
3) Stable preterm eligible for continuing KMC at the NICU
4) 16+ years of age (mother)
5) Residing within Lusaka Province with no intentions to relocate in the coming 12-18 months

Exclusion criteria:

1) Mothers who are on treatment for depression and/or anxiety
2) Mothers who did not provide informed consent
3) Underage mothers (16-17 years of age) whose parent has not provided consent for their participation in the study

Aim #3:

Inclusion criteria:

1) Parents (mothers and fathers) whose newborn have been enrolled in the study
2) Trusted family member or friend of the mother whose newborn is enrolled into the study
3) 16+ years of age (mothers and fathers)
4) 18+ years of age (family members)

Exclusion criteria:

1) Family members or parents who do not consent to study participation

Aim #4:

Inclusion criteria:

1) Fathers whose newborn has been enrolled into the study
2) 16+ years of age (father)

Exclusion criteria:

1) Fathers who are on treatment for depression and/or anxiety
2) Fathers who did not provide informed consent
3) Underage fathers (16-17 years of age) whose parent has not provided consent for their participation in the study

## Sample Size

A total of 612 study participants will be enrolled in the study for the primary outcomes drawn from a population of mothers whose newborns are admitted to WNH-UTH NICU **(Aim #1)**. Following enrolment, mother-infant pairs will be randomized into one of the 2 study arms **(Figure 1)** and analyzed as described below. A parallel two-group design will be used to test whether the Group 1 (NICU KMC and KMC ward treatment, intervention) proportion (P_1_) is different from the Group 2 (NICU KMC treatment only, control) proportion (P_2_) (H_0_: P_1_ – P_2_ = 0 versus H_1_: P_1_ – P_2_ ≠ 0). The comparison will be made using a two-sided, two-sample Z-Test with unpooled variance, with a Type I error rate (α) of 0.05. Based on our previous study, the control group proportion (P_2_) is assumed to be 0.28. To detect a proportion difference (P_1_ – P_2_) of -0.10 (or P_1_ of 0.18) with 80% power, the number of subjects needed will be 275 in Group 1 (treatment) and 275 in Group 2 (control). Adjusting for 10% loss to follow up gives 306 per group, totaling 612 subjects. The sample size was computed using PASS 2023, version 23.0.2.

The sample size for the secondary study outcomes are as follows. For the screening of developmental delays **(Aim #2)**, we will screen infants (n=612) born to mothers enrolled under aim #1. For study **Aim #3**, we will select a purposeful sampling of mothers (n=24), fathers (n=24), and family members (n=24) to undergo in-depth interviews. For **Aim #4**, we will screen fathers of the newborns enrolled into the study (n=612). Lastly, mothers from the same cohort described above will be approached to collect data for study **Aim #5**.

## Study Procedures

Per standard of care, newborns admitted to the NICU will remain there until deemed stable by the physician, at which point they will continue practicing KMC at the NICU before being admitted to the KMC ward for a minimum of 24 hours as part of the step-down care for further monitoring before being discharged home. However, based on availability at the KMC ward, stable newborns may be immediately admitted to the KMC ward once deemed stable at the NICU for the duration of their hospital stay until discharged home. Criteria used to discharge a newborn home from the KMC ward include the KMC scoring chart, clinical stability of the newborn, and their weight. Following WHO recommendations, stabilized newborns and those under routine care will begin KMC at the NICU. Potential study participants will be screened for study eligibility and enrolment at the WNH-UTH NICU.

For Aim #1, mothers will be informed about the study by study hired Research Assistants (RAs) and if they are interested and meet the study inclusion and exclusion criteria described above will be consented for study participation. Following consent, once the newborn is deemed clinically stable at the NICU by the treating physician and can continue KMC either at the NICU or at the KMC ward, will be randomized to either control or intervention study arms (described below under *Randomization*). If, on the day of hospital discharge from the NICU or the KMC ward, the treating physician decides to keep the newborn for longer based on the newborns’ health condition, the newborn will remain at the hospital until the treating physician decided to discharge the newborn.

For aims #1-2, and #4, there will be a total of up to 8 study visitations for all study participants and their infants (control and intervention arms) throughout the study. Of these, 3 will take place while mother-infant dyads are admitted to the hospital and 4 following their discharge, which are aligned with the postnatal care visits for the infant. The last – 8^th^ – study visit will combine the last clinical screening and study exit interview for all study participants – mother, father, infant (Table 1 below: *Study Visitation Schedule).* During the follow-up visits, study participants will be screened by trained RAs at the study site, in a closed and secure setting. Study procedures for aims #1, 2, and 4 are described below under *Description of Clinical Screening.*

**Table 1: Study Visitation Schedule**

| **Visit Number** | **Visit Name**  **(time frame)** | **Study Procedures** | **Clinical Screening** |
| --- | --- | --- | --- |
| **0** | Eligibility | Pre-screening | N/A |
| **1** | Enrollment | Informed Consent  Demographic Data | N/A |
| **2** | NICU Randomization | Randomization Data | N/A |
| **3** | Hospital Discharge  (KMC ward) | Demographic Data | Newborn: Clinical data |
| **4*** | Follow Up #1  (2-weeks) | Depression | Mother: EPDS & PHQ-9  Newborn: N/A  Father: EPDS & PHQ-9 |
| **5** | Follow Up #2  (6-8 weeks) | Depression | Mother: EPDS & PHQ-9  Newborn: N/A  Father: EPDS & PHQ-9 |
| **6** | Follow Up #3  (6 months) | Developmental Outcomes  & Depression | Mother: EPDS, PHQ-9 & MSPSS  Newborn: ASQ-3  Father: EPDS & PHQ-9 |
| **7** | Follow Up #4  (12 months) | Developmental Outcomes  & Depression | Mother: EPDS, PHQ-9, & MSPSS  Newborn: ASQ-3  Father: EPDS & PHQ-9 |
| **8** | Follow Up #5  (18 months)  Study Exit | Developmental Outcomes | Newborn: ASQ-3 |

*****Some study participants may undergo the 4^th^ visit while still admitted to the hospital (KMC ward).

For aim #3, study hired RAs specifically trained in qualitative research will approach potential study participants from study aims #1 and #4 and consent them for study participation under study aim #3. Additionally, the same RAs will approach trusted family members, identified through the mother and father, for participation in the IDIs as well. Following enrollment, all study participants will undergo IDIs in a safe and secure location. The RA will conduct a total of 6 IDIs six months following study enrolment, comprised of 24 purposively selected study participants from Aims #1 (mother: control arm - 12; intervention arm - 12), #4 (father: control arm – 12; intervention arm - 12), and trusted family members (family member: control arm – 12; intervention arm - 12) per group. IDIs will last approximately 1 hour and they will be conducted in the preferred language of the consented study participant. There will be only one visit for IDIs and all study participants will exit the study following the IDIs.

For aim #5, we aim to identify major cost centres, quantify all resources and attach a value to the resources required to screen for PPD women whose infants are randomized to be admitted to the KMC ward. The ingredient-based approach will be applied to estimate the average cost to the health service provider for each admitted case to the KMC and those discharged to home from the NICU. This process will help to determine unit cost for each cost centre. Costs to be identified include those associated with providing health care, costs for patients and their families, productivity costs (opportunity and overhead costs), and willingness to pay for additional services.  We will categorize costs as fixed or variable and direct or indirect costs. Direct costs refer to the value of all resources consumed to obtain medical treatment. Direct costs are further subdivided into direct medical and direct non-medical costs. Direct medical costs are the values of resources used for medical treatment such as the costs of drugs, diagnostics, procedures, laboratory tests, supplies, equipment, staff time, allowances and salaries, hospitalization, outpatient visits and overhead costs). Direct non-medical costs are the values of resources that are required for obtaining the medical treatment, such as the costs for transportation, food, lodging, etc. Indirect costs are the economic losses borne by the household due admission to the KMC. They will include 1) the patient and his caretakers’ loss of income/production; substitute labourers’ net loss of income/production; 2) productivity losses due to forgone non-market activities such as school, housework and childcare. Whenever possible, costs borne by households before reaching the hospital for KMC services will be gathered. The cost of one diagnosed case of PPD will be the sum of the costs of all services used for clinical treatment (and diagnosis) plus a fraction of buildings and overhead costs of running the health facility.

## Randomization

Mothers who meet the study inclusion and exclusion criteria described above will be consented to study participation before NICU discharge. Following consent, once the newborn is deemed clinically stable by the treating physician (defined as off oxygen support, IV fluids, feeding well) and can continue KMC either at the NICU or at the KMC ward, the study-hired RA will randomize the newborn to one of the following two study arms using block randomization **(Figure 1)**:

*Control Arm:* Study participants randomized to the control arm will remain in the NICU and then be admitted to the KMC ward for up to 2 days before discharge home by the treating physician using the KMC ward discharge criteria and the weight of the baby. During the stay at the KMC ward, per national guidelines, mothers will be guided on KMC, including breastfeeding, counseled on the importance of continuous KMC, and will be encouraged to continue practicing KMC while at home. Mother-infant dyads will be screened for PPD and neurodevelopmental delays respectively per study visitation schedule.

*Intervention Arm:* Study participants randomized to the intervention arm will be admitted to the KMC ward from the NICU immediately following randomization, where they will stay until deemed ready for discharge home by the treating physician using the KMC ward discharge criteria and the weight of the baby. Upon NICU discharge and at KMC ward admission, mothers will be guided on KMC, including breastfeeding, counselled on the importance of continuous KMC, and will be encouraged to continue practicing KMC while at the KMC ward and when discharged home. Mother-infant dyads will be screened for PPD and neurodevelopmental delays respectively per study visitation schedule.

## Description of Clinical Screening

All study participants will be screened for PPD (Edinburgh Postnatal Depression Scale [EPDS]), depression (PHQ-9), social support (Multidimensional Scale of Perceived Social Support [MSPSS]), and neurodevelopmental delays (Ages and Stages Questionnaire 3^rd^ Edition [ASQ-3]) while admitted to the hospital followed by discharge, up to 18-months of life according to the timelines described under **Figure 2**. The study-hired research associate will be trained in the use of the aforementioned screening tools by a master trainer.

**Figure 2: Schedule of Events**

Participants with depression will be referred to the Psychiatry Department for further monitoring and possible treatment. Furthermore, infants with delayed milestones will be referred to Neurology clinic at WNH-UTH. Screening at the proposed timepoint will provide us with crucial data on the prevalence of PPD, allowing us to monitor the impact of the NICU, and KMC, including disease severity and home environment, on PPD and overall depression among mothers. Detailed description of the proposed study screening tools are as follows:

*EPDS Screening*

All mothers and fathers will undergo screening for PPD using the EDPS screening tool at postnatally, at different timepoints (Appendix A). This 10-question screening tool is a low-cost and simple screening tool that has been validated for use among pregnant women in LMICs.[40] It can be used for screening of perinatal depression, beginning from antenatal period up to 6-8 weeks postnatal (PPD). Each question is scored between 0-3, the highest cumulative score being 30. Mothers who score 11+ and fathers who score 9+ will be categorized to have possible PPD and will be linked to care at the WNH-UTH Psychiatry Unit. Mothers and fathers enrolled in the study will be screened for PPD using the EPDS screening tool at 4 time points: 2 weeks, 6-8 weeks, 6 months, and 12 months.

*PHQ-9 Screening*

PHQ-9 is a 9-question patient-administered screening tool for the assessment of depression among mothers and fathers (Appendix B).[41] The tool has been used in many LMICs including Zambia.[42] Since PHQ-9 can be used repeatedly to monitor depressive symptoms among the same patient/study participant, it will be used at different time points throughout the study. Mothers and fathers enrolled in the study will be screened for depression using the PHQ-9 screening tool at 4 time points: 2 weeks, 6-8 weeks, 6 months, and 12 months. The severity scale is as follows:

1. None: 0-4
2. Mild: 5-9
3. Moderate: 10-14
4. Moderate-Severe: 15-19
5. Severe: 20-27

*MSPSS*

MSPSS is a tool for screening of perceived social support from family, friends, and significant other.[43, 44] It, a brief and easy-to-follow self-report measure, is a 12-question tool using a 5-point Likert scale (0 = strongly disagree, 5 = strongly agree) (Appendix C). It uses positive and negative phrasing to limit response set bias of agreement. Mothers enrolled in the study will be screened using the MSPSS screening tool at 6 and 12 months following enrolment. This total score can also be calculated as a mean score (divide by 12). To calculate the mean subscale scores:

- Significant Other Subscale: Sum across items 1, 2, 5, & 10, then divide by 4.
- Family Subscale: Sum across items 3, 4, 8, & 11, then divide by 4.
- Friends Subscale: Sum across items 6, 7, 9, & 12, then divide by 4.

Scores are divided into:

1. Low support: 1 to 2.9
2. Moderate support: 3 to 5
3. High support: 5.1 to 7

*ASQ-3 Screening*

ASQ is a low-cost, easy to use screening tool that measures the development of young children via screening of 5 domains – communication, problem solving, gross motor, fine motor, and personal-social. The screening tool is used between ages 1 month and five and a half years and takes around 15 minutes to complete.[45] All newborns admitted in this study will be screened for developmental delays using the ASQ tool at 6 (Appendix D), 12 months (Appendix E), and 18 months (Appendix F) of life. The cut-off score for the ASQ-3 tool different based on each domain and age and are detailed in the screening tool.

## Data Collection

Data will be collected directly from patient files and from the study participants by a study-hired Research Associate (RA) onto study-specific data collection tools. Once done, data collection forms will be reviewed by the Study Coordinator and/or PI for completeness and will then be given to the data management team. Data will then be entered by a study-hired data associate into a custom-built study database (RedCap or DHIS-2), both highly intelligent data management capturing systems that includes automated range, consistency, verification, and ID checks where possible.

Monthly monitoring reports (MMR) will be generated monthly to monitor study participant screening, enrolment, withdrawals or loss to follow-up, and follow-up throughout the study. MMRs will be generated within 1-2 weeks following the end of the previous month to provide the data entry team ample amount of time for data entry. Once generated, the report will be viewed by the Study Coordinator and PI. Additionally, monthly data audits will be followed utilizing across (data inconsistencies across different data forms) and within (data inconsistencies within the same data forms) algorithms for error resolutions.

While admitted to the hospital, all study participants (mother-infant dyads) will be monitored by the RA and government staff. Following discharge, study participants, including the fathers, will be requested to return to the study site for follow-up visits as described in Figure 2 (Aims 1, 2, and 4). Consent will be sought from the participants for either a household visit, phone call, or either of the two in case they miss a study visit. In the instance where they will be unable to return to the health facility and have consented to only a telephone call, data will be collected through a call. For Aim #3, the RA will conduct a total of 6 IDIs six months following study enrolment, comprised of 24 purposively selected study participants from Aims #1 (mother: control arm - 12; intervention arm - 12), #4 (father: control arm – 12; intervention arm - 12), and trusted family members (family member: control arm – 12; intervention arm - 12) per group. In the table below, we describe the primary and secondary outcomes for each of the five study aims (**Table 2**):

**Table 2: Study Outcomes**

| **Study Aim** | **Outcomes** |
| --- | --- |
| **Aim #1** | **Primary Outcome:**   1. Maternal PPD (6-8 weeks)   **Secondary Outcome:**   2. Maternal PPD (2 weeks, 6 months, and 12 months) 3. Duration of KMC (at the NICU, KMC ward, and at home) 4. Breastfeeding practices, including timing of initiation and duration 5. Barriers to KMC initiation |
| **Aim #2** | 1. Neurodevelopmental outcomes at 6, 12, and 18 months of life 2. Weight gain (at NICU and following hospital discharge) 3. Anthropometric measurement 4. All-cause 28-day neonatal mortality 5. Neonatal morbidity 6. Hospital readmission 7. Infant mortality 8. Infant morbidity (sepsis, diarrhea, respiratory, etc.) 9. Vaccination adherence |
| **Aim #3** | 1. Barriers and facilitators to practicing KMC at the health facility and at home 2. Social-cultural adaptation of KMC |
| **Aim #4** | 1. Paternal PPD (2 weeks, 6-8 weeks, 6 months and 12 months) 2. Time spent with newborn |
| **Aim #5** | 1. Government cost for admission to the KMC ward 2. Government cost for readmission to the NICU following randomization 3. Government costs associated with hospital readmissions within 12 months of life 4. Out-of-pocket family costs associated with hospital readmission, morbidity, and mortality |

## Data Analysis

**Aim #1:** The primary analysis will compare the proportion of maternal PPD at 6-8 weeks postnatal between the two arms using a two-sided, two-sample Z-Test with unpooled variance. We will estimate risk ratio (crude and adjusted) using a log-binomial regression model. Likelihood ratio p-value less than 0.05 will be considered statistically significant. We will conduct a multiple regression analysis to predict depression during the postpartum period using EPDS screening results collected during ANC. Data will not only be dichotomised to those with depressive symptoms and those without, and further grouped into those with anxiety and feelings of self-harm.

**Aim#2:** Using log-binomial regression model, we will estimate the effect of the intervention on risk of neurodevelopmental delays (NDD), adjusting for potential confounders. Children at risk of NDD will be classified using scores obtained from the five ASQ-3 domains compared to established cut-off. Each of the five domains (Communication, problem-solving, gross motor, fine motor, and personal and social domains) are scored between 0 – 60 with higher scores being indicative that the child’s development is on schedule. Binary secondary outcomes will be analyzed in an analogous way. However, continuous secondary outcomes will be analyzed using linear regression model, adjusted for important confounders.

**Aim #3:** A thematic analysis approach will be used for the qualitative data. Deductive coding will be applied under priori themes derived from the IDIs. Analysis will focus on identifying the barriers and facilitators of KMC at both facility and household level following hospital discharge. IDIs will be audio-recorded, transcribed, and translated into English prior to thematic analysis using NVivo 9.0 (QSR International, Australia). Analysis will begin with the familiarization process that will involve reading the transcripts, identifying key meanings related to the facilitators and barriers, and coding them under the appropriate pre-defined themes. A secondary analysis will then be done which will involve coding to identify connections between the household/community barriers/facilitators of practicing KMC at home and maternal depression.

**Aim #4:** A similar analysis approach described above under Aim #1 will be used to determine the overall prevalence of paternal PPD at 6-8 weeks and 12 months postpartum and prevalence within each arm. Furthermore, we will look at the correlation between maternal and paternal PPD overall at 12 months postpartum.

**Aim #5:** The analysis will compare the relative cost and health outcomes of two strategies by randomization - one that proposes to screen for PPD women whose infants are admitted to the KMC and the other that reflects the status quo. The cost-effectiveness analysis will be done to identify the strategy that maximizes the utility of each dollar invested in health. The effectiveness of either interventions will be measured in terms of estimated odds ratios (matched cases and controls). Potential confounding variables will be adjusted by using robust Poisson regression models. Based on this and other health service data, incremental cost-effectiveness ratios of the intervention or status quo will be measured in terms of net incremental costs per case averted, net incremental costs per death averted, and finally the net disability-adjusted life years (DALY’s) avoided by the proposed strategy as compared to the reference scenario of doing nothing (leaving the status quo).    The estimation of DALYs will consider both the reduction in morbidity (years of life lost due to disability, YLD) and mortality (years of life lost, YLL). To estimate DALYs avoided due to screening for PPD, we will use standardized weights for valuing an extra year of life regardless of the recipient’s age. Moreover, we will use the country-specific life expectancy tables’ data from WHO to estimate the number of life years saved for each age group and derive discounted life years saved using a 3% discount rate.

## Dissemination Plan

Following study completion and data analysis, results will be shared with local stakeholders through the MoH TWG meetings and published in a peer-reviewed journal within 12 months. Furthermore, study findings irrespective of results will be presented at national (NHRA conference and dissemination meetings) and internationally recognized pediatric conferences (i.e., PAS). Lastly, we will disseminate our findings through recommendations for new policies for management and screening of newly delivered mothers. Our project will inform policy change at both national and international levels.

# 8.0 Ethical Considerations

Study participation is voluntary and there will be no obligation to participate in the study. Potential study participants will be informed that the care of their newborn will not be impacted by not participating in the study. Adverse events and protocol deviations will be reported on study data forms. Unexpected, serious adverse events will be reported to all IRBs. A DSMB will monitor the study for safety and efficacy.

The study team will work hard to protect personal and health information collected in the study as described above. Similar to all other studies, there is a small risk that personal or health information could become known to others and this could cause harm to the study participants. To prevent this, research records will be kept confidential in a lockable cabinet to the maximum extent permitted by law.

## Potential Risks

There is minimal risk for study participants. Theoretical risks of this study are related to discomfort associated with possible delayed hospital discharge. There are no known risks, but only benefits, to KMC admission. Previous, smaller studies have not shown a correlation between KCM admission and any other risks.

## Potential Benefits

Potential benefits of this study are to provide stronger evidence on the impact of KMC admission on maternal PPD. Improved mental wellbeing of the mother is associated with better growth, bonding, and, possibly with better neurodevelopmental outcomes, but the means by which to achieve improved maternal PPD through admission to the KMC ward following NICU discharge has not been well studied. Furthermore, newly delivered mothers and fathers who screen positive for PPD or depression will be identified through this study and referred to Clinic 6 under the Psychiatry Unit for further evaluation. Assessment of efficacy in improving neurodevelopment and other major outcomes as well as long term growth will be studied as well. There are no direct costs to study participants. Study participants in all will receive compensation for their time in the form of cash valued at 100 ZMW and an additional 100ZMW for each follow-up visit throughout the 18-month period.

## Data Safety and Monitoring Board (DSMB)

A DSMB will be assigned to the study and is tasked to safeguard the interests of the study participants and to assist and advise the PI to protect the validity and credibility of the study. We recommend that the DSMB meet formally to review interim safety, efficacy and futility, as well as other aspects of accruing data (such as enrollment, data completeness and protocol compliance) three times during the study, after roughly 25%, 50% and 75% of subjects complete the one-months follow up. DSMB reports will be blinded, with study arms labeled as group A and group B. However, the DSMB may request to be unblinded to perform their duties. The DSMB will have full authority to suspend enrolment or stop the study altogether due to patient safety. The DSMB will debrief with the study investigators regarding their findings. A report will be written following this feedback and the research team will respond and react to recommendations made by the DSMB.

# 9.0 Timeline

All study procedures will be conducted over a 36-month period of which up to 6 months will be dedicated to receipt of regulatory approvals (UNZA BREC, NHRA, and UAB IRB). We will dedicate between 10-12 months for patient enrolment and an additional 12 months to conduct the 12-month follow-up of newborns, mothers, and fathers. Lastly, we dedicate 6 months for manuscript development and dissemination of study findings parallel to revision of national KMC guidelines. The key activities include training of study staff, patient enrolment, data entry, analysis, and dissemination of results. The detailed timeline is outlined in the following Table.

|  | **Year 1** | | | | **Year 2** | | | | **Year 3** | | | |
| --- | --- | --- | --- | --- | --- | --- | --- | --- | --- | --- | --- | --- |
|  | **Q1** | **Q2** | **Q3** | **Q4** | **Q1** | **Q2** | **Q3** | **Q4** | **Q1** | **Q2** | **Q3** | **Q4** |
| **Preparation phase** |  |  |  |  |  |  |  |  |  |  |  |  |
| Ethical approvals | X | X |  |  |  |  |  |  |  |  |  |  |
| Staff Hire and Training |  |  | X |  |  |  |  |  |  |  |  |  |
| **Implementation** |  |  |  |  |  |  |  |  |  |  |  |  |
| Patient Enrolment |  |  | X | X | X | X |  |  |  |  |  |  |
| Patient Follow-up (18 months) |  |  | X | X | X | X | X | X | X | X | X | X |
| **Monitoring and evaluation** |  |  |  |  |  |  |  |  |  |  |  |  |
| Data entry and management |  |  | X | X | X | X | X | X | X | X |  |  |
| Statistical analysis |  |  |  | X |  | X |  | X |  | X |  |  |
| Publication and dissemination |  |  |  |  |  |  |  |  |  |  | X | X |
| Revision of National Guidelines |  |  |  |  |  |  |  |  |  |  | X | X |

# 10.0 Budget

The breakdown of our proposed study budget is summarized below.

| **Category** | **Year 1**  **(Zambian Kwacha)** |
| --- | --- |
| Key Personnel | K4,566,600 |
| Medical Supplies | K63,840 |
| Patient Incentives | K286,760 |
| Administrative | K527,640 |
| Other | K544,480 |
| **Total** | **K5,989,320** |

# 11.0 Appendix

a. EPDS
b. PHQ-9
c. MSPSS
d. ASQ-3: 6-month of age
e. ASQ-3: 12-month of age
f. ASQ-3: 18-month of age
g. Data collection tools
h. CVs (Team)

- Dr. Albert Manasyan
- Prof. Anitha Menon
- Dr. Jyoti Lakwani

# References

1.	Nations, U. *Sustainable Development Goals*. 2022 [cited 2022 December 09]; Available from: <https://www.un.org/sustainabledevelopment/development-agenda/>.

2.	Shorey, S., et al., *Prevalence and incidence of postpartum depression among healthy mothers: A systematic review and meta-analysis.* J Psychiatr Res, 2018. **104**: p. 235-248.

3.	Solomon, H., et al., *Intended versus actual delivery location and factors associated with change in delivery location among pregnant women in Southern Province, Zambia: a prespecified secondary observational analysis of the ZamCAT.* BMJ Open, 2022. **12**(3): p. e055288.

4.	Roman-Galvez, R.M., et al., *Worldwide Prevalence of Intimate Partner Violence in Pregnancy. A Systematic Review and Meta-Analysis.* Front Public Health, 2021. **9**: p. 738459.

5.	Wang, Z., et al., *Mapping global prevalence of depression among postpartum women.* Transl Psychiatry, 2021. **11**(1): p. 543.

6.	Smythe, K.L., I. Petersen, and P. Schartau, *Prevalence of Perinatal Depression and Anxiety in Both Parents: A Systematic Review and Meta-analysis.* JAMA Netw Open, 2022. **5**(6): p. e2218969.

7.	Deribachew, H., et al., *prevalence of postpartum depression.* 2018.

8.	Maila, B., et al., *Prevalence and correlates of antenatal depression at Chelstone First Level Hospital in Lusaka, Zambia: a cross-sectional study.* Afr Health Sci, 2022. **22**(4): p. 334-347.

9.	Nachega, J.B., et al., *Adherence to antiretroviral therapy during and after pregnancy in low-income, middle-income, and high-income countries: a systematic review and meta-analysis.* AIDS, 2012. **26**(16): p. 2039-52.

10.	Spelke, M.B., et al., *Interpersonal therapy versus antidepressant medication for treatment of postpartum depression and anxiety among women with HIV in Zambia: a randomized feasibility trial.* J Int AIDS Soc, 2022. **25**(7): p. e25959.

11.	Stringer, E.M., et al., *Depression, pregnancy, and HIV: the case to strengthen mental health services for pregnant and post-partum women in sub-Saharan Africa.* Lancet Psychiatry, 2014. **1**(2): p. 159-62.

12.	Cleary, S., et al., *Economic burden of maternal depression among women with a low income in Cape Town, South Africa.* BJPsych Open, 2020. **6**(3): p. e36.

13.	Dadi, A.F., et al., *Epidemiology of postnatal depression and its associated factors in Africa: A systematic review and meta-analysis.* PLoS One, 2020. **15**(4): p. e0231940.

14.	Fisher, J., et al., *Prevalence and determinants of common perinatal mental disorders in women in low- and lower-middle-income countries: a systematic review.* Bull World Health Organ, 2012. **90**(2): p. 139G-149G.

15.	Slomian, J., et al., *Consequences of maternal postpartum depression: A systematic review of maternal and infant outcomes.* Womens Health (Lond), 2019. **15**: p. 1745506519844044.

16.	Lund, C., et al., *Poverty and common mental disorders in low and middle income countries: A systematic review.* Soc Sci Med, 2010. **71**(3): p. 517-528.

17.	Bonacquisti, A., P.A. Geller, and C.A. Patterson, *Maternal depression, anxiety, stress, and maternal-infant attachment in the neonatal intensive care unit.* J Reprod Infant Psychol, 2020. **38**(3): p. 297-310.

18.	Tahirkheli, N.N., et al., *Postpartum depression on the neonatal intensive care unit: current perspectives.* Int J Womens Health, 2014. **6**: p. 975-87.

19.	Wyatt, T., K.M. Shreffler, and L. Ciciolla, *Neonatal intensive care unit admission and maternal postpartum depression.* J Reprod Infant Psychol, 2019. **37**(3): p. 267-276.

20.	*Maternal depression and child development.* Paediatr Child Health, 2004. **9**(8): p. 575-598.

21.	Doukoure, D., et al., *Acceptability of the Kangaroo Mother Care at the University Hospital of Treichville in Cote d'Ivoire.* J Public Health Afr, 2022. **13**(3): p. 2165.

22.	Gebeyehu, N.A., et al., *Knowledge, attitude and practice towards kangaroo mother care among postnatal women in Ethiopia: Systematic review and meta-analysis.* PLoS One, 2022. **17**(5): p. e0265411.

23.	Muttau, N., et al., *Strengthening Kangaroo Mother Care at a tertiary level hospital in Zambia: A prospective descriptive study.* PLoS One, 2022. **17**(9): p. e0272444.

24.	Group, W.H.O.I.K.S., et al., *Immediate Kangaroo Mother Care and Survival of Infants with Low Birth Weight.* N Engl J Med, 2021. **384**(21): p. 2028-2038.

25.	Sinha, B., et al., *Effect of Community-Initiated Kangaroo Mother Care on Postpartum Depressive Symptoms and Stress Among Mothers of Low-Birth-Weight Infants: A Randomized Clinical Trial.* JAMA Netw Open, 2021. **4**(4): p. e216040.

26.	Shovers, S.M., et al., *Maternal postpartum depression: risk factors, impacts, and interventions for the NICU and beyond.* Curr Opin Pediatr, 2021. **33**(3): p. 331-341.

27.	Azale, T., A. Fekadu, and C. Hanlon, *Treatment gap and help-seeking for postpartum depression in a rural African setting.* BMC Psychiatry, 2016. **16**: p. 196.

28.	Rahman, A., et al., *Interventions for common perinatal mental disorders in women in low- and middle-income countries: a systematic review and meta-analysis.* Bull World Health Organ, 2013. **91**(8): p. 593-601I.

29.	Jawed, M., et al., *Management of maternal depression: Qualitative exploration of perceptions of healthcare professionals from a public tertiary care hospital, Karachi, Pakistan.* PLoS One, 2021. **16**(7): p. e0254212.

30.	UNFPA, *State of World Population Report 2022*. 2022, UNFPA.

31.	Rathod, S., et al., *Mental Health Service Provision in Low- and Middle-Income Countries.* Health Serv Insights, 2017. **10**: p. 1178632917694350.

32.	Rathod, S., et al., *Cultural adaptation of CBT for serious mental illness: a guide for training and practice*. 2015: John Wiley & Sons.

33.	Saraceno, B., et al., *Barriers to improvement of mental health services in low-income and middle-income countries.* Lancet, 2007. **370**(9593): p. 1164-74.

34.	McGovern, M.E., S. Rokicki, and N.E. Reichman, *Maternal depression and economic well-being: A quasi-experimental approach.* Soc Sci Med, 2022. **305**: p. 115017.

35.	Bauer, A., et al., *The lifetime costs of perinatal depression and anxiety in Brazil.* J Affect Disord, 2022. **319**: p. 361-369.

36.	Epperson, C.N., et al., *Healthcare resource utilization and costs associated with postpartum depression among commercially insured households.* Curr Med Res Opin, 2020. **36**(10): p. 1707-1716.

37.	Badiee, Z., S. Faramarzi, and T. MiriZadeh, *The effect of kangaroo mother care on mental health of mothers with low birth weight infants.* Adv Biomed Res, 2014. **3**: p. 214.

38.	Bigelow, A., et al., *Effect of mother/infant skin-to-skin contact on postpartum depressive symptoms and maternal physiological stress.* J Obstet Gynecol Neonatal Nurs, 2012. **41**(3): p. 369-82.

39.	de Alencar, A.E., et al., *Effect of kangaroo mother care on postpartum depression.* J Trop Pediatr, 2009. **55**(1): p. 36-8.

40.	Levis, B., et al., *Accuracy of the Edinburgh Postnatal Depression Scale (EPDS) for screening to detect major depression among pregnant and postpartum women: systematic review and meta-analysis of individual participant data.* BMJ, 2020. **371**: p. m4022.

41.	Ford, J., et al., *Use of the Patient Health Questionnaire (PHQ-9) in Practice: Interactions between patients and physicians.* Qual Health Res, 2020. **30**(13): p. 2146-2159.

42.	Molinaro, M., et al., *Evaluating the Relationship Between Depression and Cognitive Function Among Children and Adolescents with HIV in Zambia.* AIDS Behav, 2021. **25**(9): p. 2669-2679.

43.	Bruwer, B., et al., *Psychometric properties of the Multidimensional Scale of Perceived Social Support in youth.* Compr Psychiatry, 2008. **49**(2): p. 195-201.

44.	Zimet, G.D., et al., *Psychometric characteristics of the Multidimensional Scale of Perceived Social Support.* J Pers Assess, 1990. **55**(3-4): p. 610-7.

45.	Manasyan, A., et al., *Diagnostic accuracy of ASQ for screening of neurodevelopmental delays in low resource countries.* BMJ Open, 2023. **13**(5): p. e065076.
